# Growth Responses and Photosynthetic Indices of Bamboo Plant (*Indocalamus latifolius*) under Heavy Metal Stress

**DOI:** 10.1155/2018/1219364

**Published:** 2018-07-15

**Authors:** Abolghassem Emamverdian, Yulong Ding, Farzad Mokhberdoran, Yinfeng Xie

**Affiliations:** ^1^Co-Innovation Center for Sustainable Forestry in Southern China, Nanjing Forestry University, Nanjing 210037, China; ^2^College of Biology and the Environment, Nanjing Forestry University, Nanjing 210037, China; ^3^Bamboo Research Institute, Nanjing Forestry University, Nanjing 210037, China; ^4^Department of Agronomy and Plant Breeding, Faculty of Agriculture, Islamic Azad University, Mashhad Branch, Mashhad 94531, Iran

## Abstract

Investigating factors involved in the alleviation of the toxic effects of heavy metals (HMs) on plants is regarded as one of the important research concerns in the environmental field. The southern regions of China are severely impacted by human-induced heavy metal (HM) contamination, which poses an impediment to growth and productivity of bamboo (*Indocalamus latifolius*) plants. This necessitates the investigation of the effects of HMs on growth and physiological properties of bamboo. Therefore, the aim of the study was to evaluate some gas exchange and growth parameters in two-year-old bamboo species under HMs stress. A greenhouse-based experiment was conducted at Nanjing Forestry University, where the bamboo plant was treated with three HMs (Cu, Pb, and Zn) at four different concentrations (0, 500, 1000, and 2000 mg kg-1). The results illustrated that excessive HMs (1000 and 2000 mg kg^−1^) triggered a decline in a number of photosynthetic-related indices including the rate of photosynthesis (*μ*mol CO_2_ m^−2^ s^−1^), intercellular CO_2_ concentration (*μ*mol CO_2_ mol^−1^), conductance to H_2_O (mol H_2_O m^−2^ s^−1^), and net assimilation as well as transpiration. Morphological indices were also depressed as a result of the adverse influence of HMs, leading to decreased shoot length (10 to 73%) and reduced number of emerged plants (6 to 57%). Also, the results indicated that Pb had the greatest harmful impact on the growth indices.

## 1. Introduction

The excessive levels of heavy metals (HMs) in the soil, which mainly stem from human-induced activities, are a major threat to human health, plant productivity, and wildlife [1, 2]. HMs are viewed as one of the chief culprits for the contaminated human and animal food chains. They can accumulate in plant organs and act as a growth inhibitor by disrupting plant vital physiological and developmental processes [3, 4]. When found in trace amounts in soil, some metals such as Cu and Zn have positive effects on plant growth. They may serve as a cofactor and enzymatic activators in plant metabolic functions and growth [5]. But there are also some nonessential metals including (Cr, Cd, Pb, Hg, and Cl) that even in small quantities can exert deleterious impacts on plant growth and development [6]. The extreme concentration of metals in the soil can bring about detrimental changes to plant photosynthesis, eventually leading to the reduction of plant growth and yield [7]. Moreover, they are capable of causing oxidative stress in plants via generating, e.g., “Reactive Oxygen Species (ROS)” [8]. Heavy metals can indirectly impact plants by disrupting electron transport chain and superseding essential elements [9]. Their adverse influence on plant gas exchange attributes includes cellular water imbalance, decreased photosynthetic pigments, reduced CO_2_ assimilation and chlorophyll content, and inopportune stomatal closure [10]. Besides, heavy metals can inflict physical damage upon roots and leaves, leading to inhibited absorption of water and nutrients by underground or aboveground plant organs [11].

Bamboo plants cover large parts of China forests [12, 13]. There are 500 species at 48 genera of bamboo in China [14, 15]. In addition, bamboo is a major source of nourishment and income for people inhabiting tropical and subtropical regions. Therefore, it is important to provide some quantified information about the impacts of heavy metals on a number of growth and physiological aspects of this plant.

The objective of the study was to investigate some gas exchange parameters and growth indices in two-year-old bamboo species (*Indocalamus latifolius*) under heavy metal stress. Another aim pursued in the study was to find out if HMs in low concentrations contributed positively to the growth parameters of the bamboo plant.

## 2. Materials and Methods

### 2.1. Experimental Design and Statistical Analysis

The experiment was conducted in a completely randomized block design (CRBD) with five replications at Nanjing Forestry University greenhouse to evaluate the effects of three types of the most prevalent heavy metals found in southern area of China including (Cu, Pb, and Zn) at four different rates: 0, 500, 1000, and 2000 mg kg-1 on a two-year-old bamboo species (*Indocalamus latifolius*). Analysis of variance (ANOVA) test was computed using statistical software package R. The difference between means was compared by Tukey's test at the P>0.05 confidence level. Vertical bars in the figures indicate standard deviation (SD).

For the preexperiment stage, the two-year-old bamboo species were subjected to different concentrations of heavy metals in the spring for a period of 60 days. The specific amounts of heavy metals during the experiment period were summarized in Table 1.

### 2.2. Photosynthetic Parameters

Photosynthetic-related parameters including net photosynthetic rate (PN), conductance to H_2_O (Cond), intercellular CO_2_ concentration (Ci), and transpiration rate (Tr) were quantified using the LI-6400XT Portable Photosynthesis System (LI-COR, Lincoln, Nebraska, USA). The device is fitted with blue and red LEDs (LI-6400-02B) as a light source for the relevant photosynthetic measurements. All the leaf gas exchange properties were recorded under the sample chamber condition where photosynthetic photon flux density (PPFD) was at 1000 *μ*Mm^−2^s^−1^, leaf temperature was at 25°C, and the CO_2_ level was at 380*μ*M CO_2_ mol^−1^.

### 2.3. Growth Indices

Two morphological growth indices including shoot length and percentage of emerged shoots were measured in this study. To record shoot length, the heights of 3 to 4 of the tallest shoots in each pot were manually measured before and after the exposure of the bamboo plant to HMs stress. To determine the percentage of the emerged shoots, the number of shoots per pot was counted prior to the application of the treatments and at the end of the experiment, the difference between the initial number of shoots and surviving ones per pot/treatment was computed. Both measurements were expressed in percentage terms.

## 3. Results

### 3.1. Gas Exchange Properties (Photosynthetic Parameters)

Photosynthetic measurements revealed that high concentrations of HMs had a negative impact on the bamboo gas exchange parameters, particularly at the elevated levels (1000–2000 (mg/kg^−1^)), which led to a significant reduction in the photosynthetic indices including PN, Cond, Ci, and Tr. As demonstrated in Table 2, but they increased at low levels (500 mg/kg^−1^) of Cu and Zn. All the measured photosynthetic properties decreased at high HMs concentrations so that the HMs treatments, when applied at extreme concentrations, caused a larger decline of photosynthetic properties compared to lower levels of HMs. Overall, photosynthetic properties decreased by approximately 1.15-, 1.46-, and 1.12-fold of the control under the Cu, Pb, and Zn treatments, respectively. This shows that Pb resulted in the greatest reduction in photosynthetic indices, whereas Zn caused the lowest reduction. Moreover, across the HMs concentrations tested, 2000 mg/kg^−1^ application rate was found to exert the most pernicious impact on photosynthetic processes and 500 mg/kg^−1^ level was the concentration that caused the smallest decline in the photosynthetic parameters (under the influence of lead).

### 3.2. Effects of Cu, Pb, and Zn on the Growth Parameters

**Figure 1:**
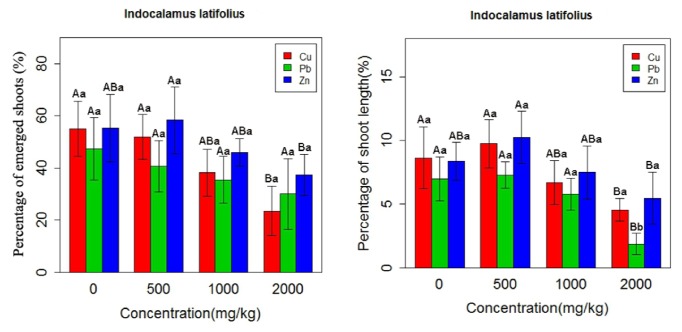
Effects of HMs on growth indices of* Indocalamus latifolius*. The capital letters are the demonstration of statistical significance between different heavy metals across various concentrations and the small letters are the demonstration of statistical significance between heavy metals in each concentration. Vertical bars represent ± SD (n=5).

Our results indicated that the lowest concentration of HMs (500 mg kg^−1^) positively contributed to the growth of bamboo species, but there was a significant reduction in the plant growth indices (shoot height and the number of emerged shoots), when HMs levels were further increased to 1000 and 2000 mg kg^−1^. The application of Cu led to an 11% increase at 500 mg/kg^−1^ and a 33% decline in growth parameters at 1000 and 2000 mg/kg^−1^. The growth parameters were increased by 4% and decreased by 40% under the 1000 and 2000 mg/kg^−1^ Pb treatments, respectively. Zinc caused a 12% increase at 1000 mg/kg^−1^ and a 23% increase at 2000 mg/kg^−1^ in growth relative to the control (Figure 1).

Shoot height measurements indicated that the low concentration of HMs encouraged the growth of shoot length. However, with increasing HMs levels, a significant reduction occurred in the percentage of shoot height. Cu, Pb, and Zn produced about 1.11-, 1.04-, and 1.18-fold increase in the shoot length over the control at 500 mg kg^−1^ but caused approximately 1.35-, 1.45-, and 1.22-fold decrease over the control at 2000 mg kg^−1^, respectively (Table 2, Figure 1).

### 3.3. Effects of HMs on the Percentage of Emerged Plants

The results of mean comparison for the effects of HMs on the percentage of emerged bamboo plants revealed the same trend for this trait as for the shoot length percentage. According to the results, the percentage of the emerged plants was about 1.06-fold higher than the control at Zn 500 mg kg^−1^, but it was about 1.31-, 1.35-, and 1.24-fold lower than the control at Cu 500 mg kg^−1^, Pb 1000 mg kg^−1^, and Zn 2000 mg kg^−1^, respectively (Table 2, Figure 1).

## 4. Discussion

### 4.1. Effects of Cu, Zn, and Pb on Photosynthetic Parameters

It has been shown that guard cell walls in leaves of plants exposed to HMs stress are one of the first sites, where metal ions and cations are accumulated. This can result in the production of H_2_O_2_ in leaf tissues [16]. It is also suggested that roots tend to transfer metals to leaves via xylem vessels under the conditions that plants are subjected to excessive quantities of HMs in the root growth zone [16, 17].

HMs often directly impinge on the structure of thylakoid membranes at chloroplast site and also on photosynthetic proteins, which thereby perniciously affect the efficiency of photochemistry properties in dark-adapted leaves and photosystem II [18]. This leads to reduced energy transmission to the photosynthetic reaction centre [19]. Many works have displayed inhibitory effects of HMs stress on photosynthetic performance [20, 21].

Our findings suggested that almost all photosynthetic properties decreased with an excess of HMs (except Cu and Zn in 500 mg/kg^−1^). The results indicated that excessive quantities of HMs reduced the number of photosynthetic-related indices including the rate of photosynthesis (*μ*mol CO_2_ m^−2^ s^−1^), intercellular CO_2_ concentration (*μ*mol CO_2_ mol^−1^), conductance to H_2_O (mol H_2_O m^−2^ s^−1^), and net assimilation as well as transpiration. These observations are in line with the findings of Chen et al. (2011) [22], where they found that Cd stress decreased the net rate of photosynthesis (Pn) and stomatal conductance (Gs) in pak choi and mustard. Similar observations are reported for sunflower and corn where net photosynthesis and transpiration were declined by HMs stress [23]. It is shown that dwindling photosynthetic rates in plants exposed to Cu excess mostly emanates from the closure of the stomatal apparatus [24].

### 4.2. Growth Indices

Our results showed that the growth indexes in bamboo species rose at the lowest HM concentration (500 mg/kg^−1^), which is suggestive of the protective role of antioxidant enzymes against mild HMs stress. In contrast, the extreme HMs levels (1000 and 2000 mg/kg^−1^) mainly led to a depressed growth in bamboo species as compared to the control (Table 3).

The observed decrease in shoot length might have occurred due to injurious effects of HMs on mesophyll cells in leaf and the stimulation of oxidative stress [25]. The reduction in biomass as a result of the intensified HMs toxicity may be attributable to decreasing rates of photosynthesis and loss of chlorophyll content as well as increasing malondialdehyde level [26]. Afshan* et al*. 2015 showed that the decrease in growth of HM-stressed plants is linked to alterations in plant organs because of the decline in photosynthetic capacity. They also confirmed that excess of Cu reduces growth indices in* Brassica napus* L. [27].

Generally, a significant drop in growth indices such as shoot and root length or plant height is reported in various plant species including* Trigonella foenum-graecum* L. grown under Cd stress [28],* Jatropha curcas *subjected to Pb excess [29],* Glycyrrhiza uralensis* seedlings exposed to Cd stress [30], tomato, canola, and Indian mustard seeds treated with Pb, Zn, and Ni excess [31],* Vicia faba* L. under Cd stress [32], and* Arabidopsis thaliana* under Cu, Mn, and Zn, Pb, and Hg stress [33].

These observations are in agreement with the results of our experiment, where it was revealed that HMs stress in high concentrations negatively affected the percentage of shoot length, resulting in decreased shoot length. Moreover, we found that the number of the emerged bamboo stands in the species used in our study was reduced when it was subjected to excessive amounts of HMs.

## 5. Conclusions and Statements

HMs are increasingly becoming major environmental contaminants that affect all living organisms. Bamboo plants are particularly exposed to this abiotic stressor in southern China, which makes it essential for researchers to conduct relevant studies to fully understand the pernicious effects of HMs on various biological and physiological aspects of bamboo. Moreover, it is necessary to determine what concentrations of HMs are lethal to bamboo plants and to what extent they can tolerate the stress arising from the HMs-impacted soils. Moreover, the assumption should be tested in bamboo plants whether or not HMs in low concentrations are beneficial to their growth and development.

The results of current work suggested that Cu, Pb, and Zn at low concentrations (500 mg kg^−1^) were associated with an increase in physiological activities and the stimulation of growth in* Indocalamus latifolius. *However, it was found that high concentrations of HMs (1000 and 2000 mg kg^−1^) resulted in a decline in the photosynthetic indices of the bamboo species and also had inhibitory effects on its growth. Besides, our results revealed that Pb had the greatest damaging impact on the gas exchange properties and growth indices in* Indocalamus latifolius*.

## Figures and Tables

**Table 1:** HM concentrations in pots.

HM concentrations	500(mg/kg^−1^)	1000(mg/kg^−1^)	2000(mg/kg^−1^)
pots	647 mg	1295mg	2591mg

**Table 2:** Photosynthetic indices of *Indocalamus latifolius* as affected by HMs stress.

HM treatment		Photosynthetic indices			
		Pn (*μ*mol CO_2_ m^−2^ s^−1^)	Cond (mol H2O m^−2^ s^−1^)	Ci (*μ*mol CO_2_ mol^−1^)	Tr (mmol H_2_Om^−2^ s^−1^)
Cu (mg kg^−1^)	0	70.2 ±1.4^Aa^	0.05± 0.02^Aa^	17.67±6.01^ABa^	1.3±0.48^ABa^
	500	75.2 ±5.2^Aa^	0.06± 0.04^Ab^	21.11± 4.5^Ab^	1.7±0.47^Ab^
	1000	63.4 ±2.5^Ba^	0.03 ± 0.007^Aa^	11.02 ± 3.9^Bb^	1.1± 0.20^ABa^
	2000	53.08 ±1.1^Ca^	0.02 ±0.01^Aa^	10.81± 4.5^Bb^	0.9± 0.41^Ba^

Pb (mg kg^−1^)	0	73.6±3.06^Aa^	0.14± 0.04^Aa^	35.93± 5.8^Aa^	2.7± 0.13^Aa^
	500	65.6± 1.4^Bb^	0.07 ± 0.04^Ba^	19.51± 6.1^ABa^	1.3± 0.25^Ba^
	1000	57.4± 1.6^Cb^	0.04± 0.01^Ba^	26.31± 6.45^Ba^	0.9± 0.25^BCa^
	2000	48.7 ±1.2^Db^	0.02± 0.01^Ba^	21.25± 6.6^Ba^	0.8± 0.42^Ca^

Zn (mg kg^−1^)	0	64.08 ±1.1^Bb^	0.07± 0.05^Aa^	16.73 ± 3.4^Aa^	1.2 ± 0.45^ABa^
	500	69.4 ± 4.2^Aa^	0.07± 0.04^Aab^	20.10 ± 6.1^Ab^	1.4± 0.264^Ab^
	1000	58.7 ±1.9^Cb^	0.05± 0.04^Aa^	16.14± 8.1^Aab^	0.9± 0.20^ABa^
	2000	50.6 ±2.08^Dab^	0.03± 0.01^Aa^	11.45± 2.5^Ab^	0.7± 0.38^Ba^

**Table 3:** Effects of different concentrations of HMs on growth indices *Indocalamus latifolius.*

HMs	Growth indices	Concentration		
		500 (mg/kg^−1^)	1000 (mg/kg^−1^)	2000 (mg/kg^−1^)
Cu	Percentage of shoot length	11↑	22↓	47↓
	Percentage emerged plants	6↓	30↓	57↓

Pb	Percentage of shoot length	4↑	17↓	73↓
	Percentage of emerged plants	14↓	25↓	36↓

Zn	Percentage of shoot length	18↑	10↓	34↓
	Percentage of emerged plants	5 ↑	17↓	32↓

Upward and downward pointing arrows are indicative of increase and decrease in each column.

## Data Availability

The data used to support the findings of this study are available from the corresponding author upon request.

## Abbreviations

HMs: Heavy metals
ROS: Reactive oxygen species.
